# Cheerfulness

**Published:** 1886-12-04

**Authors:** 


### Words of Consolation.

[Communications for this column should be addressed, "Chaplain," care of the Editor of The Hospital.]

### X.—Cheerfulness.

Hope and Cheerfulness go naturally together, just as Despondency and Melancholy. More than half the burden of sickness is removed when the patient has learnt to be cheerful, or has, at least, taken the most hopeful view of his malady. A great deal of attention is paid now in hospitals to the surroundings of patients, so that they may meet with everything possible to encourage cheerfulness. For instance, it is much better known than formerly what an influence light has upon the mind. If you want to be bright and cheerful, live in a light room and get as much sunlight as you can. If you want to be melancholy, live in the dark, and you will become dark and gloomy in spirit. Modern hospitals are built with a view to giving patients as much light as possible without dazzling them; while the pictures on the walls of the rooms, flowers, if given, the arrangement of the furniture, and many little details scarcely noticed, are all made to contribute to procure a cheerful outlook from the beds. Moreover, the nurses and attendants know very well how much their influence with the sick depends upon maintaining in themselves a cheerful disposition. No one of a melancholy temperament has any business to attempt hospital work. Gloomy faces and downcast eyes have a chilling effect anywhere; but among sick folk they are positively injurious, and do quite as much harm in their way as careless or indifferent nursing. We are grateful to think that a majority of our hospital attendants are now trained to cheerfulness. But patients, many of whom are brought in from the gloomiest surroundings, either at home or at the workshop, have no such training. Nor is it to be expected that they can be made to understand at once how much their own comfort may be enhanced by a sturdy effort to meet the cheerful aspect which is brought to bear upon them.

Your sufferings, perhaps, have been quite enough to make the bravest heart despond; or you may often be a prey to gloomy, depressing thoughts. All we ask, then, of you, for the present, is not to resist the cheerfulness of your surroundings; for that is the temptation in some cases. You see a cheerful, bright face coming towards your bed; then meet that face full, and try and look like it. You hear some encouraging words spoken when your medicine is brought, or when your food is handed to you; then try to agree with the speaker. Do not meet the encouragement with only a complaint. It has been well said: "A flower may be brought us. We may just carelessly receive it and put it in water; or we may look at it, smell it, have it by us, enjoy it, and find much instruction in it. We may be cheered, too, by the kindness which brought it." So it is with everything else. In order to arrive at cheerfulness we must sometimes begin with a struggle to meet the efforts made to cheer us.

(*To be continued.*)

